# Assessing the impact of a nutrition educational video on health-related quality of life in older home-living adults after discharge from a surgical hospital department: a randomised controlled pilot trial

**DOI:** 10.1186/s40795-025-01203-1

**Published:** 2025-11-25

**Authors:** Monica Christin Hansen, Kari Ingstad, Lisbeth Uhrenfeldt, Preben U. Pedersen

**Affiliations:** 1 Faculty of Nursing and Health Sciences, Nord University, Universitetsalleen 11, Bodø, 8026 Norway; 2 Faculty of Nursing and Health Sciences, Nord University, Levanger, Norway; 3 Department of Regional Health Research, Southern Danish University, Odense, Denmark; 4 Department of Orthopaedic Surgery, Lillebaelt Hospital, Kolding, Denmark; 5 Centre of Clinical Guidelines, Department of Clinical Medicine, Aalborg University, Aalborg, Denmark

**Keywords:** Pilot study, Health-related quality of life, Malnutrition, Disease-related malnutrition, Educational video, RCT, Knowledge translation, Nursing, Response rate

## Abstract

**Background:**

Health-related quality of life (HRQoL) is a broad multidimensional concept linked to nutritional status in older adults. This study aimed to estimate the impact of an educational video on nutrition on HRQoL among older adults during the first 3 months following their discharge from a surgical department, in preparation for a full-scale trial.

**Method:**

We conducted a single-centre randomised parallel-group pilot trial in which the participants were recruited from three surgical departments in a non-university hospital in northern Norway. A nutrition educational video was sent to the intervention group 5 days following their discharge from the hospital. Outcomes included the effect size of the eight subscales of HRQoL on a scale from 0 to 100 using the Norwegian version of the RAND 36-item short form health survey, the response rate of the Rand 36 survey and the feasibility of using block randomisation to ensure similarity between the intervention and control groups.

**Results:**

Forty-four participants were recruited over 7 months, with 24 assigned to the intervention group and 20 assigned to the control group. Thirty participants completed the study, with 17 in the intervention group and 13 in the control group. The response rate was 100%, and block randomisation ensured an equal ratio between the intervention and control groups. When comparing health-related quality of life at baseline and at 3 months, those who had seen the video (*n* = 6) seemed to have witnessed an increase in mental health by 13 ± 10, compared to 3 ± 22 in the control group.

**Conclusions:**

This study indicates that the nutrition educational video improved the mental health of disease-related malnourished older adults 3 months following their discharge from hospital. Since none of the HRQoL analyses reached statistical significance, the results are exploratory and could not be interpreted as evidence of an intervention effect. A full-scale trial is necessary to fully assess the video’s impact on HRQoL.

**Trial registration:**

NCT05860140 in ClinicalTrials.gov (retrospectively registered 17 April 2023). URL of trial: https://clinicaltrials.gov/study/NCT05860140.

## Background

Health-related quality of life (HRQoL) is a valuable indicator of overall health because it is a broad multidimensional concept that includes symptoms of health conditions or diseases, treatment side effects and functional status across social, physical and mental health life domains [1]. A relationship between the risk of malnutrition and HRQoL was observed in older, non-institutionalised people [2], with findings suggesting that an increased HRQoL was positively associated with protein, energy, lipid, magnesium, phosphorus, niacin and selenium intake [2]. Therefore, nutritional status and dietary intake have been shown to play a significant role in older adults’ satisfaction with life and experiences of health [3].

Disease-related malnutrition is a potent independent factor in poor health among the older population, represents a significant healthcare problem in the community and in hospitals [4]. Disease-related malnutrition can be a condition and something that can develop over time, resulting from inadequate nutrient intake and a disease-related systemic inflammatory response, can potentially affect all parts of the body and lead to adverse outcomes, such as prolonged hospital stay, readmission, impaired wound healing, reduced HRQoL and increased mortality [5–7]. The urgency to address this issue is underscored by the diverse barriers faced in encouraging disease-related malnourished older adults to take appropriate types and amounts of food to meet their nutritional requirements. These challenges include complex socioeconomic, physiological and environmental risk factors and the unique presentation in each individual [8, 9]. Access to home care, education level and the ability to safeguard and perform daily activities, such as food shopping and meal preparation, may affect nutrition intake [10, 11]. Simultaneously, various social, psychological, and physiological factors may contribute to reduced food intake, including illness, eating difficulties, or loss of appetite [12–14]. Older adults may also lack knowledge of the importance of a balanced diet in preventing malnutrition [15].

Transferring existing knowledge to different patient groups or individuals has traditionally been described as a method that can bridge the gap between the creation of knowledge and the users of this knowledge in the practice field [16]. Therefore, knowledge translation is defined as a ‘*dynamic and iterative process that includes synthesis, dissemination, exchange and ethically sound application of knowledge to improve the health of the population, provide more effective health services and products and strengthen the health care system*’ ([17], para 4). When videos are used to transfer knowledge, it is possible to transfer information to patients despite their geographical dispersion, which can be cost-saving for healthcare systems [16]. Videos have also been shown to improve patients’ knowledge of perioperative health and increase their satisfaction with nursing care compared to traditional patient education involving oral and paper-based instructions [18]. In similar contexts, educational videos have been shown to be effective in transferring patient knowledge in areas such as heart and cancer-related diseases [19]. However, to our knowledge, there is a lack of research on how videos on nutrition can contribute to preventing disease-related malnutrition, which our study, as preparation for a future full-scale randomised control trial (RCT), intends to fill.

This study aims to estimate the impact of a nutrition educational video on the HRQoL of older adults during the first 3 months following discharge from a surgical hospital department. We assumed that HRQoL would improve within three months. The study’s research questions are as follows:

1. How does accessing a digital educational nutritional video impact the eight subscales of health-related quality of life among older home-living adults three months after discharged from a surgical hospital department?
2. What is the response rate on the Rand-36 survey at baseline and 3 months following patients’ discharge from a surgical hospital department?
3. How feasible is block randomisation to ensure that the intervention and control groups have similar characteristics?

## Methods

### Design

This was a single-centre, two-arm pilot RCT. The paper was published according to the extension of CONSORT to pilot and feasibility trials [20].

### Participants and setting

Participants could only be included if they satisfied the following criteria: a) they were aged 65 years or older, b) they had been admitted to one of the three surgical departments in a non-university hospital in a rural area in northern Norway, c) they were home-living, d) they had access to internet facilities, e) they could read and understand Norwegian, f) they were competent to consent based on their medical history and cognitive status, g) their home address was in one of nine selected municipalities close to the hospital, g) they were due to return home directly following their discharge from the hospital or after a stay at a training centre before returning home, and, h) they had a well-known body mass index (BMI) under 24. This BMI value was chosen because a BMI below 24 can indicate a nutrition risk for older adults [16]. Potential participants could still be excluded if they were given only a liquid diet or were in the terminal phase.

Participants were recruited over 7 months, from May 2022 to January 2023. We started recruitment in one orthopaedic department. After 13 weeks, to increase the inclusion rate, we included two other surgical departments: a gastro, gynaecology, breast and surgical endocrinology department and a urology and vascular/thoracic surgery department. Collaborating with the first author, the participants were identified and gave their written consent to participate in the study to trained recruitment personnel (registered nurses) selected from the respective departments. Data collection ended in March 2023.

### Intervention

A digital educational video was developed In 2021–2022 by the authors of this article, supported by the Centre for Learning and Technology at Nord University in Norway, the patients in the target group for the study and a nutritionist. Patients in the study’s target group, who were not part of the intervention or control group, were involved in developing the intervention by reading and providing feedback on the script for the educational video to ensure that the content was relevant and user-friendly. The video presented general dietary recommendations estimated for older adults in an acute phase of illness at risk of developing malnutrition or who were already undernourished according to the *Diet Handbook* from the Norwegian Directorate of Health [21]. Since the participants in the intervention group were required to access the educational video on nutrition via email or their smartphones, the video was made accessible individually through a link sent out by the first author 5 days after the participants had been discharged from hospital. The video focused on how the participants could increase their protein and energy intake after discharge from the hospital. The viewer was instructed through speech and images to adapt the recommendations and integrate them into their daily mealtime routines. They were encouraged during the video to watch it several times. Both patients in the intervention and control groups received standard care. This included nutritional screening and the possibility of receiving general dietary advice or information from healthcare personnel, as well as follow-up by a nutritionist. None of the groups received any written or oral nutritional information or advice related to this project. Only the intervention group received the video in addition to the standard care. Table 1 presents the six-step study procedure table. No concurrent treatments or interventions took place during the study.

**Table 1:** Six-step study procedure table

Procedure in six steps	Intervention group	Control group
At baseline: Participants were informed about the study. Signed informed consent was obtained	x	x
At baseline: Demographic data were collected	x	x
At baseline: Questions about possible food related challenges were collected	x	x
At baseline: The RAND-36 survey was filled out	x	x
Five days following discharge from hospital: The nutrition educational video was sent out to the participants in the intervention group	x	
After three months: The RAND-36 survey was filled out again	x	x

### Sample size

There were no previous data on which to base a power calculation. The sample size of 44 was based on consecutive sampling over 7 months, grounded on the maximum number of participants that we could enrol due to this project’s cost and time limitations. Since this was a low-risk intervention, there was no interim analysis, nor were there stopping guidelines.

### Outcome measurements

In this trial, the impact of a nutrition educational video on HRQoL, response rate, and the feasibility of block randomisation were studied.

Patient-reported outcome measures (PROMs) are valuable and clinically significant tools for assessing treatment and rehabilitation outcomes from patients’ viewpoints [22]. In this context, the medical outcome study 36-item short form (SF-36) is considered one of the most widely used PROMs to measure HRQoL [23]. Even if questionnaires are often seen as economically advantageous, they can simultaneously be experienced as challenging for certain populations, such as older adults and children [24]. Health-related problems, such as poor vision and cognitive impairments, can affect older adults’ ability to answer questions [25]. Even if there is no established threshold for defining a high response rate, a rate of 80% or higher is considered excellent [26]. However, a low response rate can affect the quality of the survey data and the generating of reliable, validated and generalisable results [26, 27]. Therefore, when preparing for a future full-scale trial, pilot studies can deliver information about participants’ willingness to provide outcomes, making it possible to make methodological adjustments before a full-scale trial [28]. A protocol for a future full-scale trial has been published elsewhere [29]. The impact of the educational video on HRQoL was measured using the RAND-36 survey at baseline and 3 months after the patients’ discharge from the hospital. The first author personally delivered the survey to the participants at the hospital and subsequently visited them at their homes or at a preferred meeting place three months later, where the first author was available to assist with reading the questions as needed. The RAND-36 survey consists of 36 items that assess eight health concepts: physical functioning, role − physical, bodily pain, general health, vitality, social functioning, role − emotional and mental health [30]. Using the SF-36 scoring syntax, the responses to questions within each dimension were summed and converted to generate dimension scores ranging from 0 (worst possible health state) to 100 (best possible state) [23, 31, 32].

The total response rate on the RAND 36 survey at baseline and 3 months following the patients’ discharge from the hospital was measured, as were the distributions between the intervention and control groups, the participants’ ages, and the departments upon the participants’ discharge. At least 80% of the questions had to be answered for the Rand 36 survey to be considered complete. The criteria for response rate success have been estimated to be 46% [33–36]. Block randomisation is a commonly used technique in clinical trials to achieve a balance between the intervention and control groups and, at the same time, reduce bias [37] When using block randomisation, participants are matched and put into groups based on defined characteristics, and the groups are then randomly selected to be either control or intervention groups [38]. By preventing municipality health personnel from transferring knowledge from a nutrition educational video from participants in the intervention group to the participants in the control group, block randomisation can contribute to the distribution of participants based on municipalities. Testing randomisation procedures before a full-scale trial can indicate how the chosen act of randomisation will successfully balance participants’ characteristics between the intervention and control groups, thereby enabling the measurement of an intervention’s actual effect size. The first author obtained baseline information from the participants included in the study. This included demographic characteristics and possible food-related challenges to determine whether the block randomisation had ensured that the intervention and control groups consisted of participants with similar characteristics, which was considered a success criterion for proceeding with this method in a future full-scale trial.

There were no changes in the measurements during the study.

### Randomisation and blinding

The first author randomly assigned the recruited participants to the two arms using the random.org [39]. The program generated a list of 10 blocks, from which the participants were randomly assigned to the intervention or control group based on their municipality affiliation. The first author enrolled and assigned participants to the intervention based on the undertaken randomisation. The hospital’s healthcare professionals were unaware of which participants were receiving the intervention. Due to the nature of this study, the first author, who also did the data analysis, and the participants were not blinded to the study.

### Statistical analysis

Data were analysed using SPSS version 29.0.2.0 (IBM Corp., Armonk, NY, USA). Frequencies and percentages were calculated for the categorical variables. Descriptive statistics are reported as the mean, standard deviation and min–max values.

Independent t-tests were used to assess the effect size on HRQoL between the intervention and control groups (intention-to-treat [ITT] analysis) and between the video and control groups (per-protocol [PP] analysis), presented as the mean, standard deviation, min–max, 95% confidence interval (CI) and one or two-sided significance. Significance between the arms was considered at a p-value of less than 0.05. The last observation carried forward (LOCF) principle was used for missing data in the ITT analysis.

The response rate was analysed by dividing the number of completed survey responses by the total number of those surveyed and multiplying it by 100 based on the total response rate. This was presented as a percentage.

Differences between the participants’ baseline characteristics and questions about possible food related challenges were analysed using the Mann–Whitney test for ordinal data, the Pearson’s chi-square test for nominal data and the Student’s t-test for ratio interval data.

## Results

**Fig. 1:**
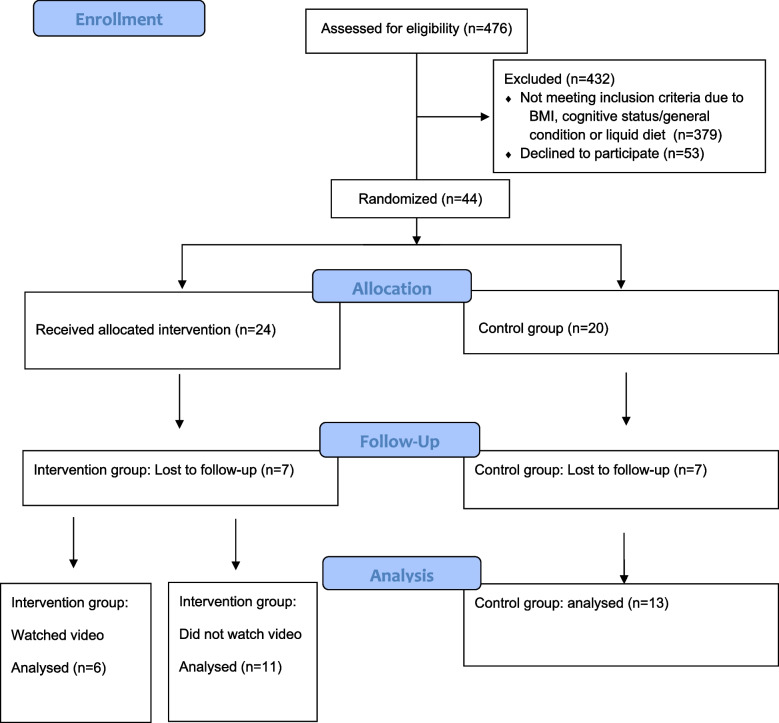
CONSORT Flow Diagram

Forty-four participants were recruited for the study during the 7-month recruitment period. Twenty-four were assigned to the intervention group and 20 to the control group. Thirty participants completed the study: 17 in the intervention group and 13 in the control group. Six participants followed the intervention by watching the video and were referred to as the video group. The participants in the video group reported having watched the video between one and four times. Figure 1 shows the study enrolment presented in a CONSORT flow diagram.

The mean age of the intervention group at baseline was 78 ± 8 years versus 77 ± 7 years in the control group. Three months after the intervention, three participants in the intervention group received home care, compared to only one in the control group. Information about the participants, including their demographic characteristics and potential food-related challenges, is presented in Table 2. No significant differences were found between the participants who completed the study and those who were lost to follow-up 3 months later. For ethical reasons, no data were collected on the cause of loss to follow-up. The participants reported no apparent adverse or harmful effects during the intervention period.

**Table 2:** Baseline characteristics and possible food-related challenges

Patients’ characteristics	Control baseline (*n* = 20) ITT	Intervention baseline (*n* = 24) ITT	P-value (two-sided)	Control and intervention baseline (*n* = 30) PP	Lost to follow-up baseline (*n* = 14)	*P*-value (two-sided)
Age, mean ± SD	77 ± 7	78 ± 8	0.53	78 ± 8	77 ± 8	0.88
(min–max)	(65–92)	(67–93)		(65–92)	(65–93)	
Gender, n (%)			0.02			0.46
Female	9 (45%)	19 (79%)		18 (60%)	10 (71%)	
Male	11 (55%)	5 (21%)		12 (40%)	4 (29%)	
Relative status, n (%)			1.83			0.18
Single	5 (25%)	9 (38%)		7 (23%)	3 (50%)	
Partner/married	15 (75%)	14 (58%)		22 (73%)	3 (50%)	
Other	0	1 (4%)		1 (3%)	0	
Education level, n (%)			0.31			0.20
Primary and secondary school	2 (10%)	5 (21%)		6 (20%)	1 (7%)	
High school	11 (55%)	1 5 (63%)		15 (50%)	11 (79%)	
College/university	7 (35%)	4 (17%)		9 (30%)	2 (14%)	
Home care, n (%)			0.33			0.49
Yes	2 (10%)	5 (21%)		4 (13%)	3 (21%)	
No	18 (90%)	19 (79%)		26 (87%)	11 (79%)	
Discharge from the following surgical department, n (%)			0.36			0.12
Orthopaedic surgery	9 (45%)	14 (58%)		14 (47%)	9 (64%)	
Stomach/intestinal, breast, gynaecology and endocrine surgical diseases	6 (30%)	3 (13%)		5 (17%)	4 (29%)	
Urology and vascular/thoracic surgery	5 (25%)	7 (29%)		11 (37%)	1 (7%)	
Responsible for shopping for food products, n (%)			0.50			0.65
The respondent	10 (50%)	11 (46%)		13 (43%)	8 (57%)	
Relatives	3 (15%)	7 (29%)		7 (23%)	3 (21%)	
Respondent in collaboration with others	7 (35%)	6 (25%)		10 (33%)	3 (21%)	
Responsible for choosing the food that is bought for the household, n (%)			0.34			0.55
The respondent	9 (45%)	16 (67%)		16 (53%)	9 (64%)	
Relatives	1 (5%)	1 (4%)		2 (7%)	0	
Respondent in collaboration with others	10 (50%)	7 (29%)		12 (40%)	5 (36%)	
Ready-made meals delivered by the municipal health service, n (%)			0.90			0.57
Yes	1 (5%)	1 (4%)		1 (3%)	1 (7%)	
No	19 (95%)	23 (96%)		29 (97%)	13 (93%)	
Experienced difficulty chewing, n (%)			0.70			0.69
Very difficult	0	2 (8%)		2 (7%)	0	
Difficult	0	2 (8%)		2 (7%)	0	
Neither difficult nor easy	2 (5%)	2 (8%)		2 (7%)	2 (14%)	
Simple	6 (30%)	4 (17%)		5 (17%)	5 (36%)	
Very simple	12 (60%)	14 (58%)		19 (63%)	7 (50%)	
Experienced difficulty swallowing, n (%)			0.58			0.09
Very difficult	0	2 (8%)		2 (7%)	0	
Difficult	1 (5%)	1 (4%)		0	2 (14%)	
Neither difficult nor easy	2 (10%)	2 (8%)		1 (3%)	3 (21%)	
Simple	5 (25%)	5 (21%)		7 (23%)	3 (21%)	
Very simple	12 (60%)	14 (58%)		20 (67%)	6 (43%)	
Experienced difficulty cutting food into portions, n (%)			0.51			0.07
Yes	2 (10%)	4 (17%)		6 (20%)	1 (7%)	
No	18 (90%)	19 (79%)		24 (80%)	13 (93%)	
Experienced difficulty moving food to the mouth, n (%)			0.49			0.55
Yes	3 (15%)	2 (8%)		4 (13%)	0	
No	17 (85%)	22 (92%)		26 (87%)	14 (100%)	
Experienced difficulty using a cup, n (%)			0.66			0.95
Yes	1 (5%)	2 (8%)		2 (7%)	1 (7%)	
No	19 (95%)	22(92%)		28 (93%)	13 (93%)	
Experienced difficulty retrieving food from the fridge, n (%)			0.85			0.15
Yes	2 (10%)	2 (8%)		4 (13%)	0	
No	18 (90%)	22 (92%)		26 (87%)	14 (100%)	
Food allergies, n (%)			0.26			0.26
Yes	3 (15%)	7 (29%)		7 (23%)	3 (21%)	
No	17 (85%)	17 (71%)		23 (77%)	11 (79%)	

### Intervention impact on HRQoL

HRQoL was measured using the Rand-36 survey, which includes eight domains, each on a scale from 0 (poor health) to 100 (good health). First, none of the analyses reached statistical significance; the results are exploratory and could not be interpreted as evidence of an intervention effect. Three months after the intervention, the ITT analysis using Student’s t-test showed no significant changes in effect size. Even though the result was not significant (*p* = 0.27), the value of mental health increased especially positively in the intervention group (4 ± 10) compared with the control group (2 ± 17). The scores for bodily pain were higher, indicating less pain in the intervention group than in the control group (11 ± 17 vs. 2 ± 31), as were the scores for social functioning (9 ± 23 vs. 1 ± 28) and role − emotional (11 ± 47 vs. 7 ± 45). However, the ITT analysis found that physical functioning and role − physical changed more in the control group than in the intervention group (10 ± 15 vs. 3 ± 20, and 9 ± 28 vs. −5 ± 36, respectively). In comparing those who viewed the video (*n* = 6) and those in the control group (*n* = 13), a paired t-test analysis using Student’s t-test found that mental health scores increased more in the video group than in the control group (13 ± 10 vs. 3 ± 22); however, this difference was not statically significant (*p* = 0.15). The video group also increased more in terms of bodily pain (9 ± 20) and social functioning (4 ± 10) compared to the control group (3 ± 38 and 2 ± 36, respectively). However, the PP analysis found that physical functioning increased more in the control group than in the video group (15 ± 17 vs. 4 ± 15, *n* = 6). General health also increased more in the control group (7 ± 16) compared to the video group (2 ± 10). Finally, role − emotional increased in the control group compared to the video group (10 ± 57 vs. 0 ± 56). None of the mean effect size changes between the video and control groups in the PP analysis were significant. Due to the study’s sample size, we found that large variations in individual participants’ effect sizes had a significant impact on the groups’ overall scores. Based on the combined physical scale, the power calculation presented in [29] showed that we would need to recruit 276 participants for the full-scale study. No ancillary analysis was performed.

Table 3 presents the comparisons of HRQoL at baseline and 3 months after the intervention within and between the intervention, control and video groups.

**Table 3:** Health-related quality of life at baseline and after 3 months

**Variable**	**Control** **baseline** **(*n* = 20)** **ITT**	**Control** **after 3 months** **(*n* = 20)** **ITT**	**Control** **after 3 months** **(*n* = 13)** **PP**	**Intervention** **baseline** **(*n* = 24)** **ITT**	**Intervention** **after 3 months** **(*n* = 24)** **ITT**	**Intervention** **after 3 months** **(*n* = 17)** **PP**	**Video** **baseline** **(*n* = 6)** **PP**	**Video** **after 3 months** **(*n* = 6)** **PP**
Physical functioning								
(0–100) mean ± SD	52 ± 30	62 ± 26	51 ± 34	56 ± 31	59 ± 29	64 ± 26	71 ± 19	75 ± 22
(min–max)	(0–95)	(0–95)	(0–95)	(5–100)	(5–100)	(20–100)	(50–100)	(45–95)
Role-physical								
(0–100) mean ± SD	25 ± 41	34 ± 42	31 ± 48	36 ± 39	31 ± 38	35 ± 42	49 ± 41	50 ± 42
(min–max)	(0–100)	(0–100)	(0—100)	(0–100)	(0–100)	(0–100)	(0–100)	(0–100)
Bodily pain								
(0–100) mean ± SD	51 ± 32	53 ± 31	57 ± 33	51 ± 32	61 ± 31	71 ± 26	72 ± 29	81 ± 22
(min–max)	(0–100)	(10–100)	(0–100)	(0–100)	(12–100)	(22–100)	(31–100)	(51–100)
General health								
(0–100) mean ± SD	55 ± 24	59 ± 25	53 ± 23	57 ± 22	57 ± 21	58 ± 20	75 ± 7	77 ± 14
(min–max)	(10–92)	(15–100)	(10–82)	(0–87)	(15–92)	(15–92)	(67–87)	(57–92)
Vitality								
(0–100) mean ± SD	39 ± 26	43 ± 23	45 ± 27	48 ± 24	50 ± 26	57 ± 24	67 ± 19	73 ± 14
(min–max)	(0–90)	(0–90)	(0–90)	(0–90)	(0–95)	(13–95)	(35–90)	(55–90)
Social functioning								
(0–100) mean ± SD	62 ± 30	63 ± 32	63 ± 31	61 ± 32	70 ± 28	73 ± 26	77 ± 28	81 ± 19
(min–max)	(0–100)	(0–100)	(0–100)	(0–100)	(0–100)	(0–100)	(25–100)	(50–100)
Role–emotional								
(0–100) mean ± SD	48 ± 49	55 ± 45	38 ± 51	51 ± 44	63 ± 45	75 ± 42	67 ± 42	67 ± 52
(min–max)	(0–100)	(0–100)	(0–100)	(0–100)	(0–100)	(0–100)	(0–100)	(0–100)
Mental health								
(0–100) mean ± SD	69 ± 25	70 ± 22	71 ± 24	72 ± 22	76 ± 21	83 ± 11	77 ± 13	90 ± 7
(min–max)	(8–96)	(8–96)	(12–96)	(16–96)	(16–100)	(68–100)	(56–92)	(80–100)

The effect sizes presented as the results for changes in HRQoL between-group analyses are shown in Table 4.

**Table 4:** Intention to treat and per-protocol analysis of change in health-related quality of life

Variable	Control (*n* = 20)	Intervention (*n* = 24)	P-value (one- sided)	Control (*n* = 13)	Video (*n* = 6)	P-value (one- sided)
Physical functioning			0.11			0.09
(0–100) mean ± SD	10 ± 15	3 ± 20		15 ± 17	4 ± 15	
(min–max)	(−5–50)	(−60–30)		(−5–50)	(−15–30)	
CI: 95%	[3.426–16.574]	[−5.002–11.002]		[5.759–24.241]	[−8.002–16.002]	
Role–physical			0.09			0.26
(0–100) mean ± SD	9 ± 28	−5 ± 36		13 ± 35	1 ± 44	
(min–max)	(−25–100)	(−100–75)		(−25–100)	(−50–75)	
CI: 95%	[−3.271–21.271]	[−19.403–9.403]		[−6.026–32.026]	[−5.802–19.802]	
Bodily pain			0.12			0.35
(0–100) mean ± SD	2 ± 31	11 ± 17		3 ± 38	9 ± 20	
(min–max)	(−79–68)	(−20–42)		(−79–68)	(−16–41)	
CI: 95%	[−11.586–15.586]	[4.199–17.801]		[−17.657–23.657]	[−7.003–25.003]	
General health			0.29			0.25
(0–100) mean ± SD	4 ± 13	2 ± 13		7 ± 16	2 ± 10	
(min–max)	(−15–32)	(−25–31)		(−15–32)	(−15–15)	
CI: 95%	[−1.697–9.697]	[−7.201–3.201]		[−1.698–15.698]	[−34.207–36.207]	
Vitality			0.30			0.48
(0–100) mean ± SD	5 ± 18	2 ± 16		7 ± 22	7 ± 16	
(min–max)	(−35–30)	(−45–35)		(−35–30)	(−20–25)	
CI: 95%	[−2.889–12.889]	[−4.401–8.401]		[−4.959–18.959]	[−5.802–19.802]	
Social functioning			0.16			0.44
(0–100) mean ± SD	1 ± 28	9 ± 23		2 ± 36	4 ± 10	
(min–max)	(−38–75)	(−25–63)		(−38–75)	(0–25)	
CI: 95%	[−11.271–13.271]	[−0.202–18.202]		[−17.569–21.569]	[−4.002–12.002]	
Role-emotional			0.38			0.36
(0–100) mean ± SD	7 ± 45	11 ± 47		10 ± 57	0 ± 56	
(min–max)	(−100–100)	(−100–100)		(−100–100)	(−100–67)	
CI: 95%	[−12.722–26.722]	[−7.804–29.804]		[−20.985–40.985]	[−44.809–44.809]	
Mental health			0.27			0.15
(0–100) mean ± SD	2 ± 17	4 ± 10		3 ± 22	13 ± 10	
(min–max)	(−36–48)	(−12–24)		(−36–48)	(0–24)	
CI: 95%	[−5.450–9.450]	[−0.001–8.001]		[−8.959–4.959]	[4.998–21.002]	

### Response rate

Out of 44 possible participants at baseline, 42 answered all the questions in the Rand 36 survey, while two participants answered 97% of the questions. Three months later, similar tendencies were observed among 30 possible participants; the majority (*n* = 29) answered all of the survey questions, while one participant answered 94%. Since all the participants had answered a minimum of 80% of the questions in the Rand 36 survey, the overall response rate was 100% at baseline and 3 months later, considered as positive in relation to going ahead with a full-scale study. Essentially, the participants filled in the survey themselves, although some received assistance in reading the questions from the first author, who was present while the surveys were being completed. However, the response rate did not seem to be influenced by the participants’ ages; diagnosis groups, based on the departments they were admitted from; or whether they were in the intervention or control group.

### Feasibility of block randomisation

The intervention group consisted of a baseline of 79% women and 21% men, compared to 45% women and 55% men in the control group (*p* = 0.02). This showed a significant difference in gender between the intervention and control groups. However, we can assume that the gender difference between the groups was related to random variations in the study’s sample size and was not clinically significant in this context. No other significant differences were found between the intervention and control groups based on the participants’ baseline characteristics or potential food-related challenges, as described in Table 2. Apart from gender, which could be due to random variations, we can thus determine that block randomisation succeeded in ensuring that the intervention and control group participants had similar characteristics at baseline for the intervention. Incidentally, no significant differences were found between those who completed the study (*n* = 30) and those who were lost to follow-up (*n* = 14).

## Discussion

This randomised controlled pilot trial aimed to estimate the impact of a nutrition education video on the HRQoL of older home-living adults during the first 3 months following discharge from a surgical hospital department.

To measure the impact of a nutrition education video on HRQoL, we used the Norwegian RAND-36, a self-administered survey with the same items as the SF-36 [30]. SF-36 has been recognised as a reliable and valid tool to use in different populations and settings, such as older adults in primary care, patients with coronary artery disease and patients with rheumatoid arthritis [40–42]. Another strength of using the SF-36 as a measurement tool for measuring HRQoL is that it provides a broad dimension of the physical and mental health changes that can occur with age, illness or alterations in functional status [43]. Thus, the RAND-36 provided a relatively extensive dimension of how the participants’ health changed during the 3 months of the intervention.

Since none of the analyses of HRQoL reached statistical significance, the results are exploratory and could not be interpreted as evidence of an intervention effect. However, our study revealed that the parameter of mental health increased substantially among the participants who had seen the nutrition educational video compared to the control group after 3 months (intervention: 13 ± 10, control: 3 ± 22). Even if the results were not significant, our findings are supported by an earlier RCT focusing on the daily intake of oral nutritional supplements, which found that participants consuming a placebo had lower quality of life scores than the intervention group, with the least squares mean being significant for the mental component summary at day 90 (−4.23 [−7.75, −0.71]; *p* = 0.019) [44]. As defined by the World Health Organization ([45], para 1), mental health is ‘*a state of mental well-being that enables people to cope with the stresses of life, realise their abilities, learn well and work well, and contribute to their community*’*.* Therefore, an individual’s mental health underpins one’s ability to make decisions, build relationships and create the world in which one lives [45]. Thus, good mental health is considered a prerequisite for rehabilitation following hospital discharge. For example, orthopaedic trauma has been shown to have significant psychological, social and physical effects on patients (Mattsson, 1975, cited in 46). Indicators of anxiety, post-traumatic disorder or depression have been reported in up to 56% of patients during their postoperative recovery [46]. Nevertheless, for most surgical patients, the main focus is physical therapy and rehabilitation in the postoperative setting to help them retain their strength and function [46]. If future full-scale trials demonstrate that delivering nutritional knowledge through educational videos can support patients’ mental health, this approach may represent a valuable component of broader health promotion strategies. Further research is therefore warranted to evaluate the effectiveness of video-based nutrition education, as our forthcoming full-scale trial aims to investigate.

Our study revealed relatively low changes in the impact of the seven other subscales on the Rand 36 survey. However, since this was a pilot study, appropriate power and sample size were not calculated. It has been advisable not to test the hypothesis for a future main study in a pilot study, and the results of a pilot study must, therefore, be reported cautiously [47–49]. Our results, thus, only indicate the impact of the nutrition educational video on HRQoL. Due to the low number of participants, we suggest that further research be conducted with greater power to establish the total effect size of the nutrition educational video on HRQoL. Moreover, the relationship between HRQoL and nutrition status is generally complex, as it is influenced by factors such as digestive problems, swallowing or chewing problems or sense perceptions, such as loss of taste or reduced appetite, that affect food intake, thereby influencing activities of daily living, mobility and mental health [50]. Overall, the participants who completed our study reported having little difficulty regarding possible food-related challenges. For example, 80% of these participants found it easy or very easy to chew and 90% found it simple or very simple to swallow. We found no significant differences between those who completed the study and those who were lost to follow-up. Incidentally, it was impossible to obtain data on the causes of the losses to follow-up in this study. Therefore, we assume that possible food-related challenges were not a reason for the losses to follow-up or did not influence the participants’ HRQoL results.

One possible limitation of the study was that offering a nutrition education video encompasses the use of a specific format (video), as well as the information provided about nutrition in the video. Interpreting the results can thereby be challenging, as it may be unclear whether the observed difference is due to the video format or the information itself. To clarify this, it may be necessary to conduct a future study where one group of participants is given the information in writing and another group is given the video to compare whether it affects the results.

A strength of this study is that the response rate on the Rand 36 survey was 100%, indicating that the participants were motivated to answer the survey regardless of their affiliation to the intervention or control group or in terms of illness or age, although the participants’ ages ranged from 64 to 93 years. Earlier, Jacobsen and colleagues [34] received an overall response rate of 36% when measuring health-related quality of life in a Norwegian sample (*N* = 6165) using the Rand-36 survey, but the response rate of the oldest adults (> 70 years) was only 27%. In this study Jacobsen, Bye [34], the surveys were distributed via email. Response rates tend to be higher in face-to-face interviews than from e-mail or online surveys, which may have an 11–12% lower turnout [51]. Our study received a response of 100%, and we believe that the fact that the first author was present during the answering of the survey, including being available both to answer questions about the completion of the forms and to read out questions, when necessary, contributed to our high response rate. This method will, therefore, be repeated in future main trials to ensure that the findings are representative of the study population.

The act of randomisation contributes to balancing the participants’ characteristics between the groups so that any differences in outcomes can be attributed to the study intervention [52]. Although there was a significant difference in gender between the intervention and control groups, a strength of this study was that we did not find any other indications that block randomisation contributed to differences between the intervention and control groups, which could have influenced the study outcome. Traditionally, older men have tended to use technology, such as the internet, to a greater extent than older women, but this has changed in recent years [53]. There are now trends that the differences have evened out as older women are using the internet to a greater extent, for example, for social contact with family and friends [53]. We therefore do not consider that gender might have influenced the outcomes of the digital intervention in this study. Because block randomisation was chosen to reduce bias in cases where healthcare personnel inadvertently shared information from the video with the control group, this study showed that 13% of the participants received home care 3 months after the intervention. Therefore, it does not appear that block randomisation was a disadvantage for the implementation of the study and can thus be prolonged to the main trial.

To the best of our knowledge, this is the first pilot study to investigate the estimated impact of a nutrition educational video on HRQoL among older adults during the first 3 months following discharge from a surgical department. Thus, we were unable to find earlier research to establish the effect size of this study. The recommended appropriate sample size in pilot studies seems to vary from 10–12 per group to 60–75 per group, depending on the study’s primary objective [54]. Due to limited time and resources, a limitation of this study was the sample size of 44, of which only six participants in the intervention group succeeded in watching the educational video. The non-significant results can be attributed to the insufficient sample size and the challenges with opening the link to the educational video. In a feasibility study [55] in connection to this pilot study we found that the reason for the participants having challenges with opening the link was seen in connection with; to be anxious about opening unknown links, not understanding how to open the link, not remembering to receive the video link, or not prioritizing to open the link because they had so much other thing to handle after the hospital stay. These findings are supported by earlier research in a similar Norwegian population, which found that older adults could experience obstacles, such as a lack of basic instructions and knowledge on how to use technology, and that using technology could feel like a demanding and overly rushed process [56]. As a result, older adults may experience a sense of dependency on others when using new technology, which could lead to feelings of helplessness [56].

As a part of preparation for a future full-scale trial, a strength of this pilot study is that it showed that, in a future full-scale trial, the participants must be educated about watching the educational video before leaving hospital. Text messages and phone calls can be used to remind participants to watch the video and contact the research group if problems arise while watching it. A full-scale trial should be conducted in the future to measure the full effects of the intervention. As part of the future full-scale trial, a feasibility study was conducted in relation to this pilot [55], and a protocol for the future full-scale trial was developed [29].

The real effect of the nutrition education video on older adults' HRQoL and potential pathways with food intake and nutritional status will be investigated in a future main trial. Here, recruiting more participants would help increase the sample size, thereby facilitating the obtaining of significant results that can confirm or refute the main trial hypothesis.

## Conclusion

This study indicates that an educational video on increasing protein and energy intake after a hospital stay may increase mental health in relation to HRQoL in older adults. However, a full-scale trial is needed to measure the full effectiveness of this video in terms of HRQoL. If standardised videos on nutrition can contribute to better HRQoL, then they can improve the quality of services for patients and relieve the workload of healthcare personnel. This can contribute to finding solutions to a major challenge in healthcare services in large parts of the world.

## Data Availability

All investigators involved in this study had full access to the data. The datasets used are available from the corresponding author upon reasonable request.

## Abbreviations

BMI: Body mass index
HRQoL: Health-related quality of life
ITT analysis: Intention-to-treat analysis
LOCF: Last observation carried forward
PP analysis: Per-protocol analysis
PROMs: Patient-reported outcome measures
RAND-36: RAND 36-item short-form health survey
RCT: Randomised control trial
SD: Standard deviation
SF 36: 36-Item short-form survey
